# Proteomics-based vaccine targets annotation and design of multi-epitope vaccine against antibiotic-resistant *Streptococcus gallolyticus*

**DOI:** 10.1038/s41598-024-55372-3

**Published:** 2024-02-28

**Authors:** Peng Chao, Xueqin Zhang, Lei Zhang, Aiping Yang, Yong Wang, Xiaoyang Chen

**Affiliations:** 1 Department of Cardiology, People’s Hospital of Xinjiang Uygur Autonomous Region, Urumqi, China; 2 Department of Nephrology, People’s Hospital of Xinjiang Uygur Autonomous Region, Urumqi, China; 3 Department of Traditional Chinese Medicine, People’s Hospital of Xinjiang Uygur Autonomous Region, Urumqi, China

**Keywords:** Immunoinformatics, Reverse vaccinology, Pan-genome, Multi-epitope vaccine, Computational biology and bioinformatics, Immunology, Structural biology

## Abstract

*Streptococcus gallolyticus is* a non-motile, gram-positive bacterium that causes infective endocarditis*. S. gallolyticus* has developed resistance to existing antibiotics, and no vaccine is currently available. Therefore, it is essential to develop an effective *S. gallolyticus* vaccine. Core proteomics was used in this study together with subtractive proteomics and reverse vaccinology approach to find antigenic proteins that could be utilized for the design of the *S. gallolyticus* multi-epitope vaccine. The pipeline identified two antigenic proteins as potential vaccine targets: penicillin-binding protein and the ATP synthase subunit. T and B cell epitopes from the specific proteins were forecasted employing several immunoinformatics and bioinformatics resources. A vaccine (360 amino acids) was created using a combination of seven cytotoxic T cell lymphocyte (CTL), three helper T cell lymphocyte (HTL), and five linear B cell lymphocyte (LBL) epitopes. To increase immune responses, the vaccine was paired with a cholera enterotoxin subunit B (CTB) adjuvant. The developed vaccine was highly antigenic, non-allergenic, and stable for human use. The vaccine's binding affinity and molecular interactions with the human immunological receptor TLR4 were studied using molecular mechanics/generalized Born surface area (MMGBSA), molecular docking, and molecular dynamic (MD) simulation analyses. *Escherichia coli (*strain K12) plasmid vector pET-28a ( +) was used to examine the ability of the vaccine to be expressed. According to the outcomes of these computer experiments, the vaccine is quite promising in terms of developing a protective immunity against diseases. However, in vitro and animal research are required to validate our findings.

## Introduction

*Streptococcus gallolyticus* is a non-motile, gram-positive bacterium that was formerly known as Streptococcus bovis. It is a phenotypically diverse strain of bacteria from the Lancefield Group D Streptococci^1^. Although frequently found in microflora, 2.5-15% of it is found in a healthy person's gastrointestinal system and develops into an opportunistic pathogen that causes a variety of diseases, including colon cancer, infectious endocarditis, septicemia, and meningitis^2^.

Infective endocarditis incidence has increased significantly globally over the past 20 years^3–5^. Streptococcal infections contributed significantly to 2.6-7 occurrences of endocarditis per 100,000 people recorded each year, with incidences of 31% in European nations, 39% in South America, 17% in North America, and 32% in the rest of the world^5^. This condition primarily affects older patients, with a median age of 58^6^. *S. gallolyticus* endocarditis is more likely to occur in those who consume fresh dairy products or raw meat, have a history of hepatic illnesses, have weakened immune systems, and have comorbid conditions such as rheumatic diseases and diabetes mellitus^7^.*S. gallolyticus* attempts to injure the endocardium when there is a primary infection, a metabolic disease, or an immune-compromised state. Following this injury, the loss of fibrin and platelets causes the formation of a thrombus. After thrombus development, the bacteria travel through the thrombus into the bloodstream. *S. gallolyticus* contains virulence traits that enable it to enter the bloodstream paracellularly and adhere to the endocardium, the collagen-rich surface of the damaged heart valve, without triggering a major immune response. This bacterium multiplies and forms a biofilm after adhering to the endocardium, inflaming the heart's lining and leading to endocarditis^8,9^.

Commonly prescribed antibiotics for infectious endocarditis include Gentamycin, Penicillin G, and streptomycin. Alternative options for individuals with penicillin allergies include vancomycin and gentamicin-related Ceftriaxone^10^. A costly surgical intervention may be required for patients who have a chronic fever and are resistant to medicinal therapy. *S. gallolyticus* is penicillin-resistant, and one strain of *S. gallolyticus* has also been proven to be tetracycline-resistant^11^. Hence, the development of an effective endocarditis treatment strategy, innovative therapeutic targets, and effective vaccines are urgently needed.

**Figure 1:**
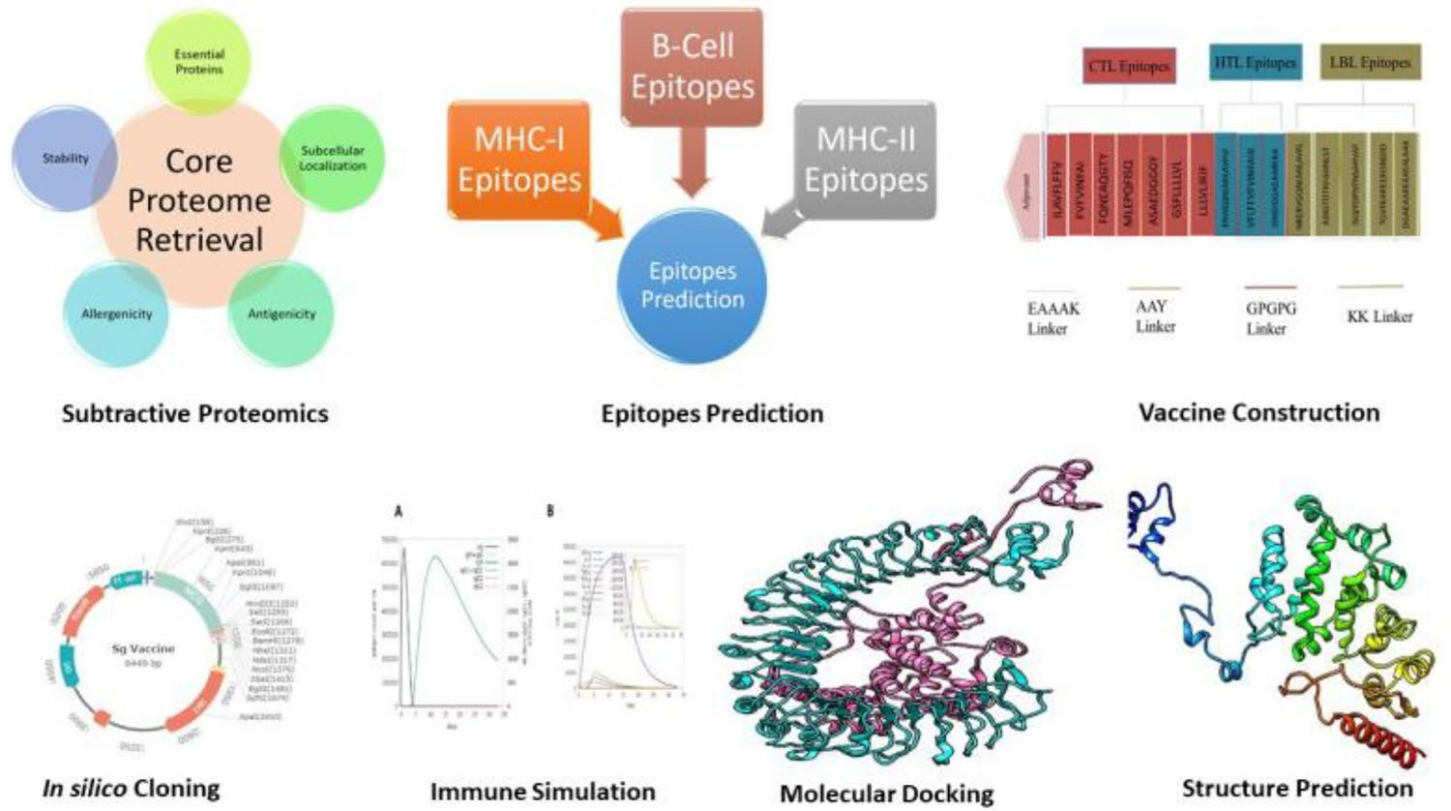
Graphical abstract.

Vaccines offer protection against infectious diseases^12^. Vaccines stimulate the immune system, and their immunogenicity is critical in achieving protection against specific pathogens. It takes a long time for a vaccine candidate to successfully develop to the point of commercial release because of the numerous clinical trial phases that must be completed and the substantial financial commitment required.However, the vaccine's success rate can be bleak at times. In such a scenario, the immunoinformatics approach is beneficial in reducing the time required for vaccine development when compared to conventional approaches. There are examples of successful vaccine candidate development using *in silico* approaches^13–19^. Numerous computer methods have been developed for quick identification, such as subtractive genomic and core genomic approaches, which enable us to find the core essential genes that are not homologous to the human genome^18–21^. This study's goal is to employ *in silico* techniques to discover potential vaccine targets by connecting the proteome and genetic data of the *S. gallolyticus* species. In this study, the core proteome of seven strains of *S. gallolyticus* was analyzed to find potential vaccine candidates. *S. gallolyticus* proteins have been targeted to forecast B cell, IFN, and T cell epitopes. Subunit vaccines were developed by selecting conserved and high antigenic epitopes and then combining them with linkers and adjuvants (Figure 1). The vaccine's physicochemical properties, allergenicity, structural features, and antigenicity were investigated using online tools. Furthermore, docking among the TLR-4 and vaccine was performed. The advanced polyprotein formation was ultimately investigated via in-silico cloning. This study could open the path for the creation of dynamic and effective vaccines that comprise a special blend of several antigenic peptides produced from *S. gallolyticus* to suppress *S. gallolyticus* infection.

## Material and methods

### Core proteomes identification

In this study, seven *S. gallolyticus* strains with complete assembly levels were considered for pan-genome analysis and their proteomes were downloaded from NCBI.. Humans were considered hosts. Their information is presented in Additional File 1: Table S1. The core proteome analysis was then performed on the proteomes using the Bacterial Pan Genome Analysis Tool (BPGA)^22^to identify conserved proteins shared by all strains to select potential vaccine candidates.

### Vaccine candidate prioritization

It is believed that essential proteins are crucial for cell viability. The core proteome of *S. gallolyticus* strains was uploaded to the Geptop 2.0 server to find essential proteins. The cutoff value for essentiality was set at 0.24. Geptop can be used for any bacterial species with a sequenced genome^23,24^. Essential proteins were uploaded to BlastP using an e-value of 10-4, and query coverage and identity of more than 70 % and 30% respectively against the human proteome to find non-homologous protein sequences of *S. gallolyticus*^25^. Proteins having no similarity with the human proteome were considered non-homologous proteins. For genome analysis and genome annotation in bacterial infections, it is crucial to predict protein subcellular localization because these proteins may serve as the main targets for drugs or vaccines^26^. The BUSCA server was employed to forecast non-homologous protein subcellular localization^27^. Furthermore, their antigenicity and allergenicity were evaluated using the Vaxijen 2.0 and AllerTop tools, respectively^28,29^. The molecular weight (MW) and stability were calculated using the ProtParam program^30^. Further, the TMHMM server was utilized to forecast transmembrane helices^31^. Proteins having 0 transmembrane helices that are non-allergenic, antigenic, and stable were identified as prospective vaccine candidates.

### Prediction of epitopes and selection phase

T-cell (HTL and CTL) and B-cell epitopes were predicted from the potential vaccine candidates. The MHC-I and MHC-II binding tools of the Immune Epitope Database (IEDB) were used to predict CTL and HTL epitopes, respectively^32^. The consensus approach was applied to a provided protein sequence in FASTA format. The human species was picked as the origin of all alleles. The epitopes with a consensus score of < 2were chosen based on their capacity to bind. ABCPred server was used to forecast linear B cell epitopes. To further evaluate the anticipated epitopes, ToxinPred, VaxiJen (v2.0), the MHC class I immunogenicity, and Allergen FP1.0 servers were employed^33–36^. Finally, the IFNepitope web server was utilized to screen HTL epitopes that induce IFN-gamma^37^

### Population coverage analysis

IEDB's web-based population coverage analysis tool was utilized to determine the population distribution of predicted epitopes. This tool provides the frequency of HLA alleles in 78 various population subgroups across 11 various geographic locations^38,39^.

### Designing and structural analysis of vaccine

Due to their small size, epitopes typically aren't able to elicit an immune response when used alone in a vaccine. In order to activate the innate and adaptive immune systems, they require a carrier that contains strong immunostimulatory adjuvants^40^. Thus, an EAAAK linker was used to join the vaccine's N terminal to the cholera enterotoxin subunit B (CTB) adjuvant. In order to replicate the immunogen's ability to function as a separate immunogen and produce higher antibody concentrations than a single immunogen, linkers are necessary in the vaccine's design^41^. The AAY, GPGPG, and KK linkers were used to join CTL, HTL, and LBL epitopes, respectively.The 3Dpro program from the SCRATCH suite was utilized to forecast the 3D structure of the vaccine^42^. The structure was refined further using an online galaxy refinement server^43^. The refined structure was further validated through the RAMPAGE server, Verify 3D, PROSAweb server, and ERRAT server^44–47^.

### Post analysis of vaccine

Allergenicity testing can predict the vaccine's capacity to produce allergic responses. Hence, the AllerTop server was used^30^. VaxiJen v2.0 was utilized to analyze structural antigenicity^28^. The SCRATCH suite's SOLpro server was used to determine the vaccine's solubility^48^. To determine the nature and stability of the vaccine, stereochemical characteristics were predicted utilizing the ProtParam server^30^. Finding the secondary structure required the use of the SOPMA server^49^. Conformational and linear B cell epitopes of the vaccine were forecasted through the Ellipro server^50^.

### Disulfide engineering

Disulfide engineering is a one-of-a-kind technique for inserting disulphide links within the target protein structure. Disulphide bonds include covalent interactions that aid in protein stability and the study of protein dynamics and interactions. To improve stability, the vaccine structure was uploaded to the Disulphide by Design (DbD) 2.12 tool^51^. A series of parameters was used to identify potential residue pairs (2.2 energy value and -87 to + 97 chi3 value), which were then altered with a cysteine residue.

### Molecular docking analysis

For the protein-protein interaction, the HADDOCK-v-2.4 server was used^52^. HADDOCK was used to create a model of the biomolecular complexes. The docked complexes were visualized using PyMOL-v.1.3^53^. PDBsum, an online service, was utilized to evaluate interactions between docked complexes^54^. Docking analysis was used to evaluate the binding potency of vaccine constructs with immune cell receptor (TLR4). Molecular dynamics simulations were then performed on docked complexes with high binding affinities.

### Molecular dynamics simulation

Using the Schrodinger suit's Desmond software, MD simulations were run at 100 ns^55^. MDS was employed to analyze a dynamic interaction of protein-protein complexes generated by molecular docking. The complex was preprocessed, then optimized and minimized. The forcefield OPLS_2005 was employed in the minimization procedure^56^. Transferable intermolecular potential with a three point (TIP3P) solvent model along with an orthorhombic box (10 × 10 × 10) was added using the system builder tool^57^. The model was neutralized by adding counterions as necessary, and 0.15 M of NaCl salt was added to simulate the physiological state. Conditions for the NPT ensemble were 1 atm pressure and 300 K temperature. Prior to the simulation, the complex was relaxed. The trajectory was saved at 50 ps for simulation results analysis.

### MMGBSA analysis

Binding free energies are frequently calculated using the empirical equation-based MMGBSA approach. The accuracy of this approach exceeds that of many molecular docking score functions^58,59^. The ante-MMPBSA.py module examined and produced the complex's initial prompt files. Using the receptor, complex, and vaccine energies variation, free energy was determined:

$$\Delta {\text{G}}_{{{\text{bind}}}} = \left( {\Delta {\text{G}}_{{{\text{complex}}}} } \right) - \left( {\Delta {\text{G}}_{{{\text{receptor}}}} + \Delta {\text{G}}_{{{\text{vaccine}}}} } \right)$$

### Codon optimization and in silico cloning

The increased expression rates may be a result of a unique codon adaptation method designed for *E. coli* K12. The main sequence for the subunit vaccine was expressed more frequently in the host cell type *E. coli* K12 using the technique, and it was then submitted to the JAVA Codon Adaptation Tool (Jcat) for modification^60^. The restriction enzyme cleavage site, the RHO independent transcription termination site, and the bacterial ribosome binding site were all avoided. Using the GenSmart design tool, the optimum nucleotide sequence for the developed multi-subunit vaccine was cloned between restriction sites within the *E. coli* pET-28a (+) vector^61^.

### Immune simulation

The C-ImmSim server was utilized to run *in silico* immune simulations to test the immunological response and immunogenicity of the *S. gallolyticus* vaccine^62^. The lowest time interval between two vaccine doses that have been scientifically advised is one month. One-time, 84-time, and 168-time step variables were developed for three inoculations that were given at one-month intervals since a one-time step equals 8 hours of daily life. All other triggering parameters retained their preset levels.

## Results

### Core proteome retrieval and vaccine candidate prioritization

Seven well-known pathogenic strains of *S. gallolyticus* were taken into consideration for the development of a vaccine. Core proteome analysis was performed, and 9576 proteins were retrieved. After being uploaded to the Geptop server for essential protein prediction, 325 of the 9576 proteins were determined to be essential genes. Blast P was used to find essential proteins that are not similar to humans. A total of 138 non-homologous proteins were discovered out of 325 important proteins. The prediction of subcellular localization was utilized to determine how certain proteins fulfill their roles. 121 of the total 138 targets were anticipated to be cytoplasmic and were therefore omitted from the research. The remaining 17 cytoplasmic membrane proteins were considered for future analysis. These proteins were also tested for antigenicity, allergenicity, and stability. Six proteins have been identified as antigenic, allergenic, and stable. Additionally, their transmembrane helices were evaluated. Two antigenic, allergenic, and stable proteins having 0 transmembrane helices were selected as vaccine candidates: penicillin-binding protein and ATP synthase subunit.

### Epitopes selection phase

CTL, B-cell, and HTL epitopes of specific antigenic proteins were forecasted. A large number of epitopes were forecasted, and the top seven CTL epitopes for vaccine formulation were chosen because they were non-toxic, immunogenic,antigenic, and non-allergenic (Table 1). Similarly, the top three non-allergenic, antigenic, immunogenic, IFN-gamma inducing, and non-toxic HTL epitopes were chosen for vaccine design (Table 2). Likewise, the top five antigenic, immunogenic, non-allergenic, and non-toxic LBL epitopes were picked for vaccine design (Table 3). Toxicity of the final epitopes were predicted and mentioned in Additional File 1: Table S2.

**Table 1:** Finalized CTL Epitopes for multi-epitope vaccine designing.

Protein	Epitopes	Alleles	Position	Immunogenicity	Antigenicity	Allergenicity
P1	ILAVFLFFV	HLA-A*02:02 HLA-A*02:06 HLA-A*69:01 HLA-A*01:01	32–40	0.3	2.6	Non-allergen
	FVFVINFAI	HLA-A*68:02 HLA-C*14:02 HLA-B*39:01 HLA-A*29:02 HLA-B*07:02 HLA-B*27:20	39–47	0.3	2.9	Non-allergen
	FQNEAQGTY	HLA-B*15:02 HLA-A*30:02 HLA-B*35:01 HLA-A*01:01 HLA-A*29:02 HLA-B*27:05	281–289	0.07	0.6	Non-allergen
	MLEPQFISQ	HLA-A*02:16 HLA-A*03:01	478–486	0.03	1.0	Non-allergen
	ASAEDGGGY	HLA-A*30:02 HLA-A*01:01 HLA-B*15:17 HLA-A*26:02 HLA-A*26:01 HLA-B*58:01	559–567	0.2	2.1	Non-allergen
P2	GSFLLLLVL	HLA-B*27:05 HLA-B*40:02 HLA-B*39:01 HLA-B*27:20 HLA-B*40:01	18–26	0.02	0.9	Non-allergen
	LLLVLIKIF	HLA-B*15:01 HLA-A*32:01 HLA-A*24:03 HLA-B*08:01	22–30	0.04	1.1	Non-allergen

**Table 2:** Finalized HTL Epitopes for multi-epitope vaccine designing.

Protein	Epitopes	Alleles	Antigenicity	Position	Immunogenicity	IFN-inducer
P1	ERVGQNLMILAVFLF	HLA-DRB1*13:01 HLA-DRB1*11:02 HLA-DRB1*11:21 HLA-DRB1*13:05	1.4	24–38	0.03	Positive
	VFLFFVFVINFAIII	HLA-DRB1*01:02 HLA-DRB1*07:03 HLA-DPA1*02:01/DPB1*01:01 HLA-DRB1*04:08 HLA-DRB1*15:06	2.8	35–49	0.8	Positive
P2	ISNDIDGAEAAREKA	HLA-DQA1*03:01/DQB1*03:02 HLA-DQA1*04:01/DQB1*04:02 HLA-DRB1*03:01 HLA-DRB3*01:01 HLA-DRB1*04:05	1.2	46–60	0.4	Positive

**Table 3:** Finalized LBL Epitopes for multi-epitope vaccine designing.

Protein	Epitopes	Position	Score	Antigenicity	Immunogenicity	Allergenicity
P1	NRERVGQNLMILAVFL	22	0.62	1.0	0.11	Non-allergen
	AIIIGTDTKFGHNLST	46	0.93	0.6	0.16	Non-allergen
	ISQIYDPNTNSARVAT	484	0.97	0.6	0.11	Non-allergen
P2	TGVFKAREEKISNDID	36	0.72	0.5	0.05	Non-allergen
	DGAEAAREKAEALAAK	51	0.71	1.1	0.3	Non-allergen

### Population coverage analysis

**Figure 2:**
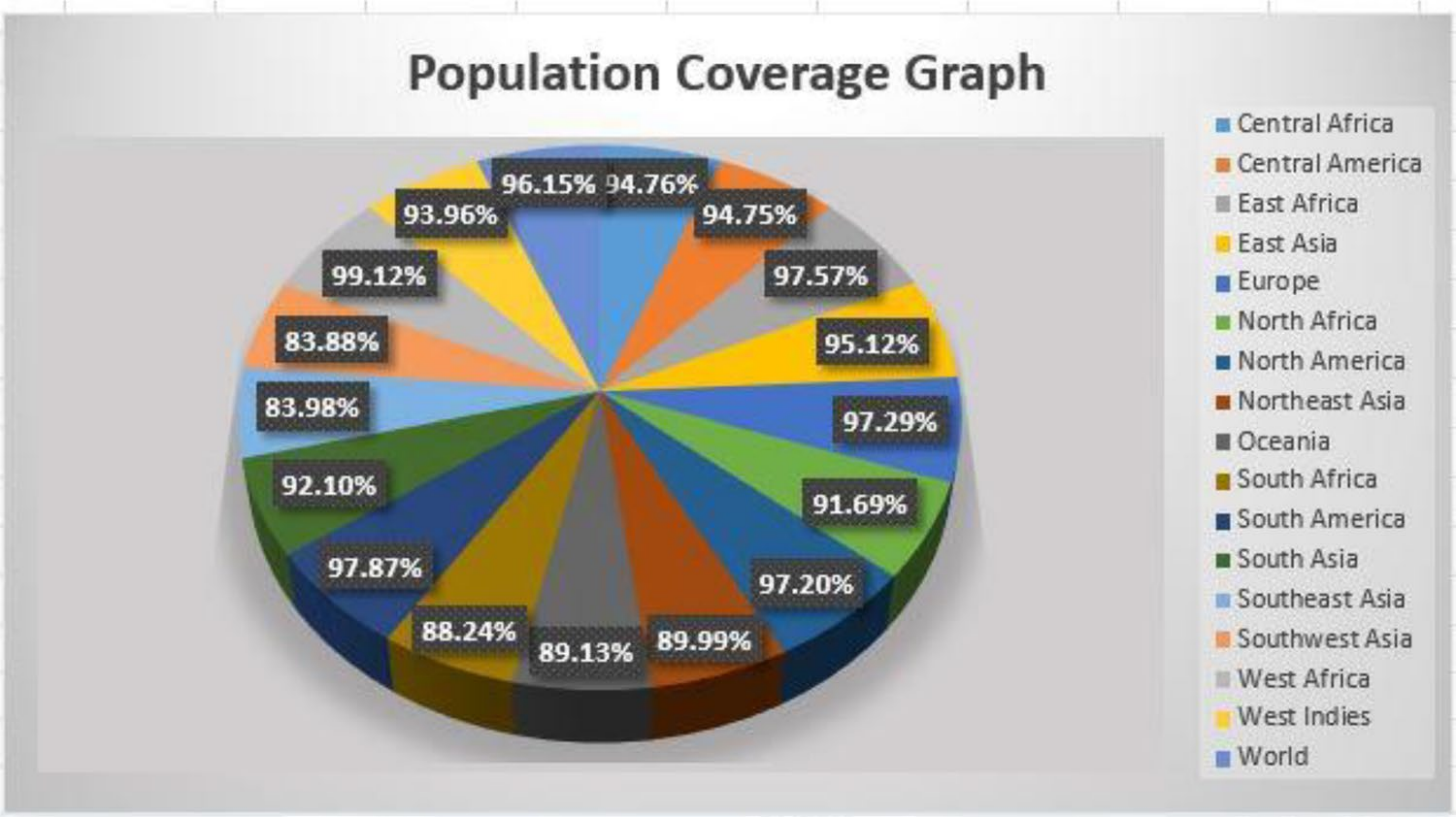
*S. gallolyticus* vaccine population coverage map, 96.15% of the global population is covered.

A population coverage analysis was conducted on specific epitopes (CTL and HTL) used in the development of the multi-epitope vaccine. The chosen epitopes, according to the statistics, encompass 96.15% of the global population. Figure 2 shows the population of various countries.

### Designing and structural analysis of vaccine

**Figure 3:**
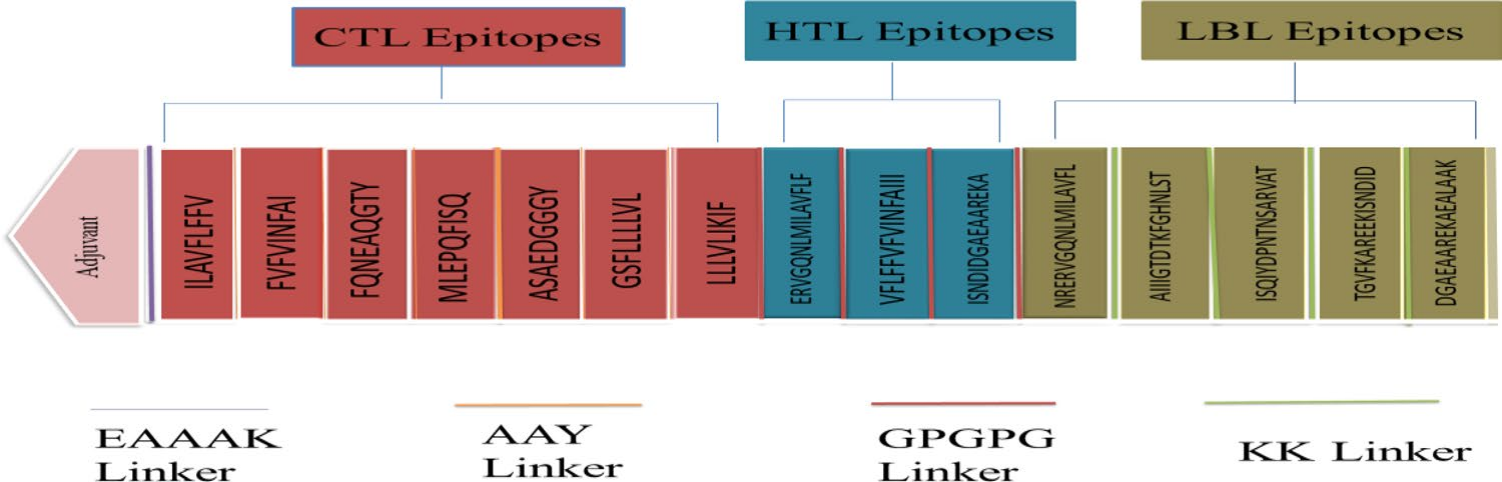
The *S. gallolyticus* vaccine construct is shown schematically.

**Figure 4:**
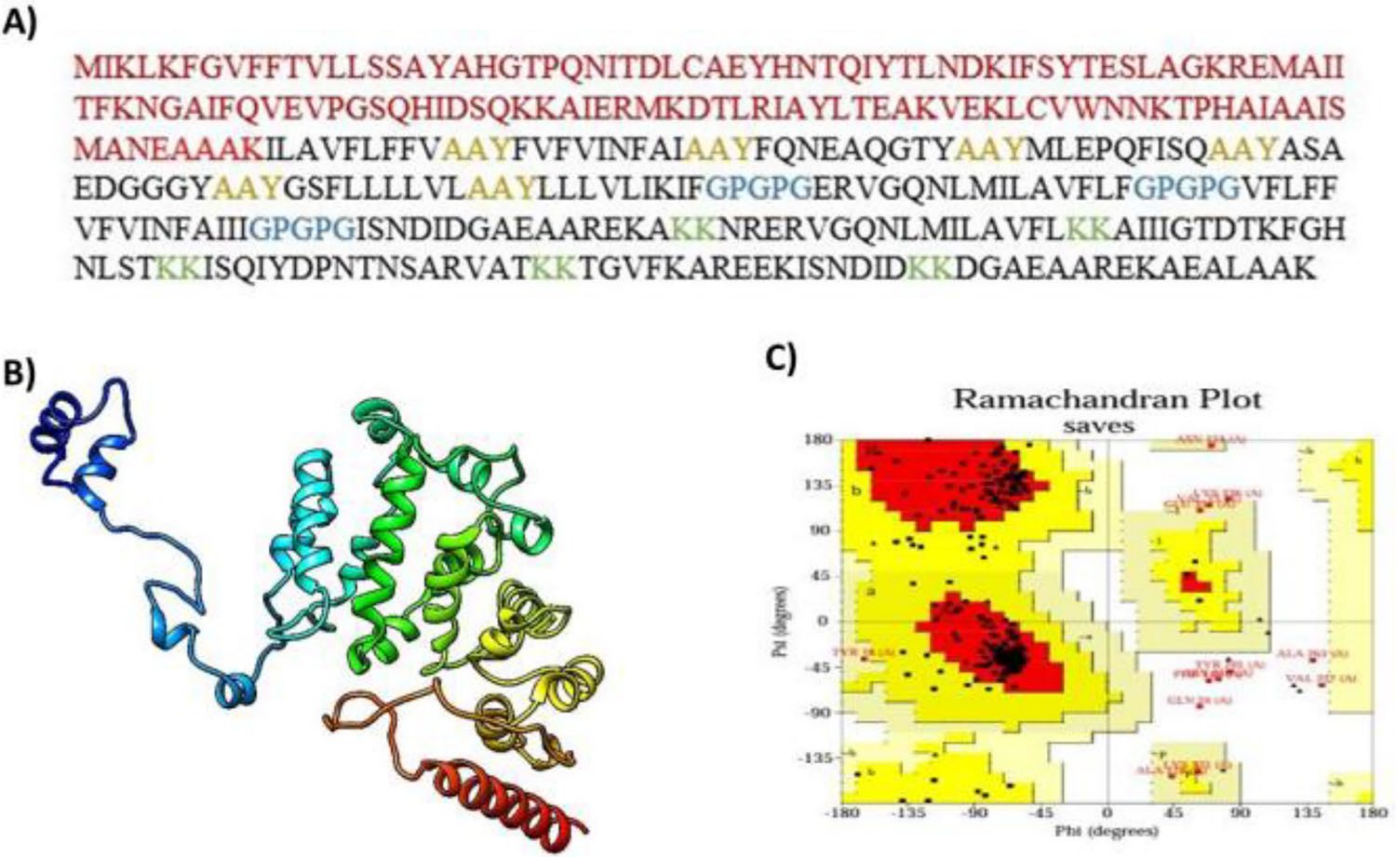
Vaccine Construct Prediction and Validation. (**A**) The adjuvant sequence is shown in maroon. The EAAAK linker in red, the epitope sequence is black, the AAY linker in golden, the GPGPG connectors in blue, and the KK linkers in green (**B**) 3D structure of *S. gallolyticus* vaccine (**C**) Ramachandran plot analysis of the structure which shows that 9.0% and 86.9% of all amino acids were positioned in the permitted and most favorable regions, respectively, whereas 2.2% were found in the disallowed region.

To construct the vaccine, 7 CTL, 3 HTL, and 5 B-cell epitopes were coupled with the appropriate adjuvant and linkers. The adjuvant, cholera enterotoxin subunit B (236 amino acids), was linked to the N-terminus of the vaccine via the EAAAK linker. Following that, AAY linkers, GPGPG linkers, and KK linkers were employed to connect CTL, HTL, and B-cell epitopes, respectively (Figure 3). The final vaccine has 360 amino acids (Figure 4A).

The 3Dpro server was used to anticipate the 3D structure of the vaccine (Figure 4B). Additionally, the Galaxy refine server was used to refine it. The model's accuracy was confirmed by a Ramachandran plot study, which showed that 9.0% and 86.9% of all amino acids were positioned in the permitted and most favorable regions, respectively, whereas 2.2% were found in the disallowed region (Figure 4C). Further analysis revealed that the final vaccine structure, with a quality factor of 77.632 and a Z-score of -3.06 does not contain any poor rotamers.

### Post analysis of vaccine

The ProtParam program was used to analyze the stereo-chemical properties of the generated structure, which indicated that the vaccine structure had an isoelectric point of 9.11 and a molecular weight of 39371.79 daltons, demonstrating the basic nature of the constructed vaccine structure. There are 39 positive amino acids (arginine, lysine) and 32 negative amino acids (glutamic acid, aspartic acid). The proposed structure's instability index is 21.74 and the aliphatic index is 99.33, both of which show that the vaccine's host-designed structure is stable. The GRAVY value was 0.199, showing that the vaccine structure is hydrophobic. The half-life of this vaccine design was estimated to be 30 hours in mammals (in vivo), > 20 hours in yeast (in vivo), and > 10 hours in E. coli (in vivo). Based on the SOLpro server results, it was predicted that the proposed vaccine structure would be soluble with a probability of 0.684246, allowing for simple access to the host. The structure of non-allergenic, non-toxic, and antigenic vaccine was also predicted. According to Vaxijen, the antigenicity result was 0.8738. SOPMA was used to investigate the vaccine's secondary structure. Among the 359 amino acids, 174 are involved in the formation of the α-helix (48.33% of the overall vaccine structure), 80 are engaged in the extended strands (22.22%), and 80 are involved in the coils (22.22%) formation. The Ellipro server predicted 6 continuous/linear B cell epitopes (Table 4) and 6 discontinuous/conformational B cell epitopes in the vaccine model (Table 5).

**Table 4:** Linear B cell epitopes predicted from vaccine.

No	Chain	Start	End	Peptide	Number of residues	Score
1	A	1	70	MIKLKFGVFFTVLLSSAYAHGTPQNITDLCAEYHNTQIYTLNDKIFSYTESLAGKREMAIITFKNGAIFQ	70	0.829
2	A	331	360	KAREEKISNDIDKKDGAEAAREKAEALAAK	30	0.742
3	A	147	170	NFAIAAYFQNEAQGTYAAYMLEPQ	24	0.689
4	A	294	325	IGTDTKFGHNLSTKKISQIYDPNTNSARVATK	32	0.629
5	A	124	141	NEAAAKILAVFLFFVAAY	18	0.617
6	A	234	239	PGVFLF	6	0.561

**Table 5:** Conformational B cell epitopes predicted from vaccine.

No	Residues	No. of residues	Score	3D structure
1	A:M1, A:I2, A:L4, A:K5, A:F6, A:G7, A:V8, A:F9, A:F10, A:T11, A:V12, A:L13, A:L14, A:S15, A:S16, A:A17, A:Y18, A:A19, A:H20, A:G21, A:T22, A:P23, A:Q24, A:N25, A:I26, A:T27, A:D28, A:L29, A:C30, A:A31, A:E32, A:Y33, A:H34	33	0.935	
2	A:N35, A:T36, A:Q37, A:I38, A:Y39, A:T40, A:L41, A:N42, A:D43, A:K44, A:I45, A:F46, A:S47, A:Y48, A:T49, A:E50, A:S51, A:L52, A:A53, A:G54, A:K55, A:R56, A:E57, A:M58, A:A59, A:I60, A:I61, A:T62, A:F63, A:N65, A:G66, A:A67, A:I68, A:F69	34	0.735	
3	A:I294, A:G295, A:T296, A:D297, A:T298, A:K299, A:F300, A:G301, A:H302, A:D314, A:P315, A:N316, A:T317, A:N318, A:S319, A:A320, A:R321, A:V322, A:A323, A:T324, A:K325, A:F330, A:K331, A:A332, A:R333, A:E334, A:E335, A:K336, A:I337, A:S338, A:N339, A:D340, A:I341, A:D342, A:K343, A:K344, A:D345, A:G346, A:A347, A:E348, A:A349, A:A350, A:R351, A:E352, A:K353, A:A354, A:E355, A:A356, A:L357, A:A358, A:A359, A:K360	52	0.687	
4	A:V138, A:A140, A:Y141, A:F144, A:V145, A:N147, A:F148, A:A149, A:I150, A:A151, A:Y153, A:F154, A:N156, A:E157, A:A158, A:Q159, A:G160, A:T161, A:Y162, A:A163, A:A164, A:Y165, A:E168, A:P169, A:Q170, A:F210, A:P234, A:G235, A:V236, A:F237, A:L238, A:F239, A:F242	33	0.664	
5	A:M282, A:N303, A:L304, A:S305, A:T306, A:K307, A:K308, A:I309, A:S310, A:Q311, A:I312, A:Y313	12	0.629	
6	A:A85, A:N124, A:E125, A:A126, A:A127, A:A128, A:K129, A:I130, A:L131, A:A132, A:V133, A:F134, A:L135, A:F136, A:F137	15	0.594	

### Disulfide engineering

**Figure 5:**
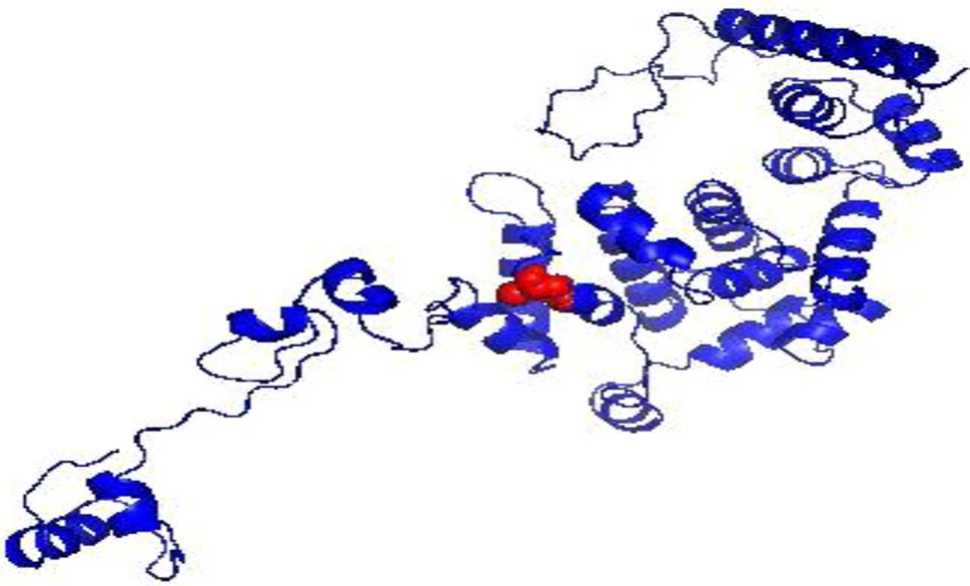
Disulphide engineering of vaccine structure to enhance stability. The red color represents one mutated pair selected based upon energy and X_3_ value.

The vaccine sequence was examined using the Disulfide by Design 2.13 server, and a total of twenty potential residue pairs that could form a disulfide bond were found. After accounting for the bond energy and the X_3_ parameters, one pair of residues (113THR-116ALA) was chosen because its results fit the requirements specified earlier (Figure 5).

### Molecular docking

**Figure 6:**
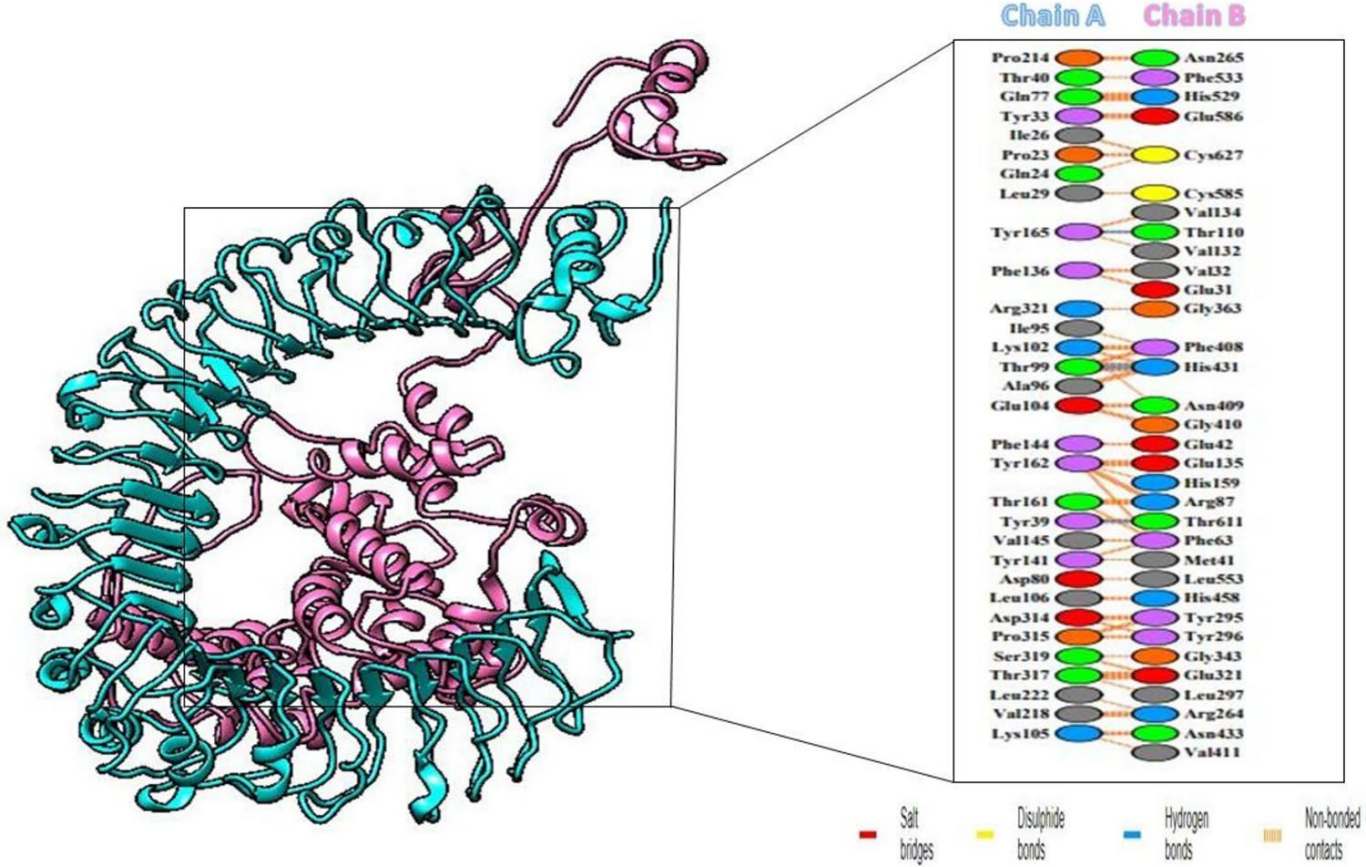
TLR-4 receptor and vaccine docked together. TLR4 is displayed in cyan, while the vaccine is displayed in pink. Red lines between the residues represents salt bridges, yellow line represents disulphide bonds, blue line represents hydrogen bonds, and orange line represents non-bonded contacts.

To elicit an effective immune response, a vaccine must have a significant affinity for the host's immunological receptors, notably toll-like receptors. The protein-protein docking investigation between the human TLR4 and the proposed vaccine was performed using the HADDOCK v. 2.4 server. According to the docking research, the vaccine and TLR4 exhibit strong binding interactions. The TLR4-vaccine binding score was 698.1 +/- 21.8 kcal/mol. The statistics from the docking assay are depicted in Table 6. In Figure 6, a docked complex is displayed. Cyan is used to symbolize TLR4, while pink is used to represent the vaccine. The PDBsum server was utilized to obtain in-depth knowledge of the binding interactions between TLR4 and vaccine in order to create the interactions map among the docked-complexes. Four hydrogen bonds between the vaccine and TLR4 in the range of 2.91Å were detected.

**Table 6:** Docking statistics of vaccine-TLR4 complex.

Docking Statistics	Score
HADDOCK score	698.1 ± 21.8
Cluster size	4
Electrostatic energy	−124.2 ± 14.6
Van der Waals energy	−116.5 ± 12.1
Restraints violation energy	9029.8 ± 257.3
Desolvation energy	−63.5 ± 1.3
Z-score	−0.7
Buried surface area	3644.0 ± 266.2
RMSD from the overall lowest-energy structure	13.5 ± 0.1

### MD simulation

**Figure 7:**
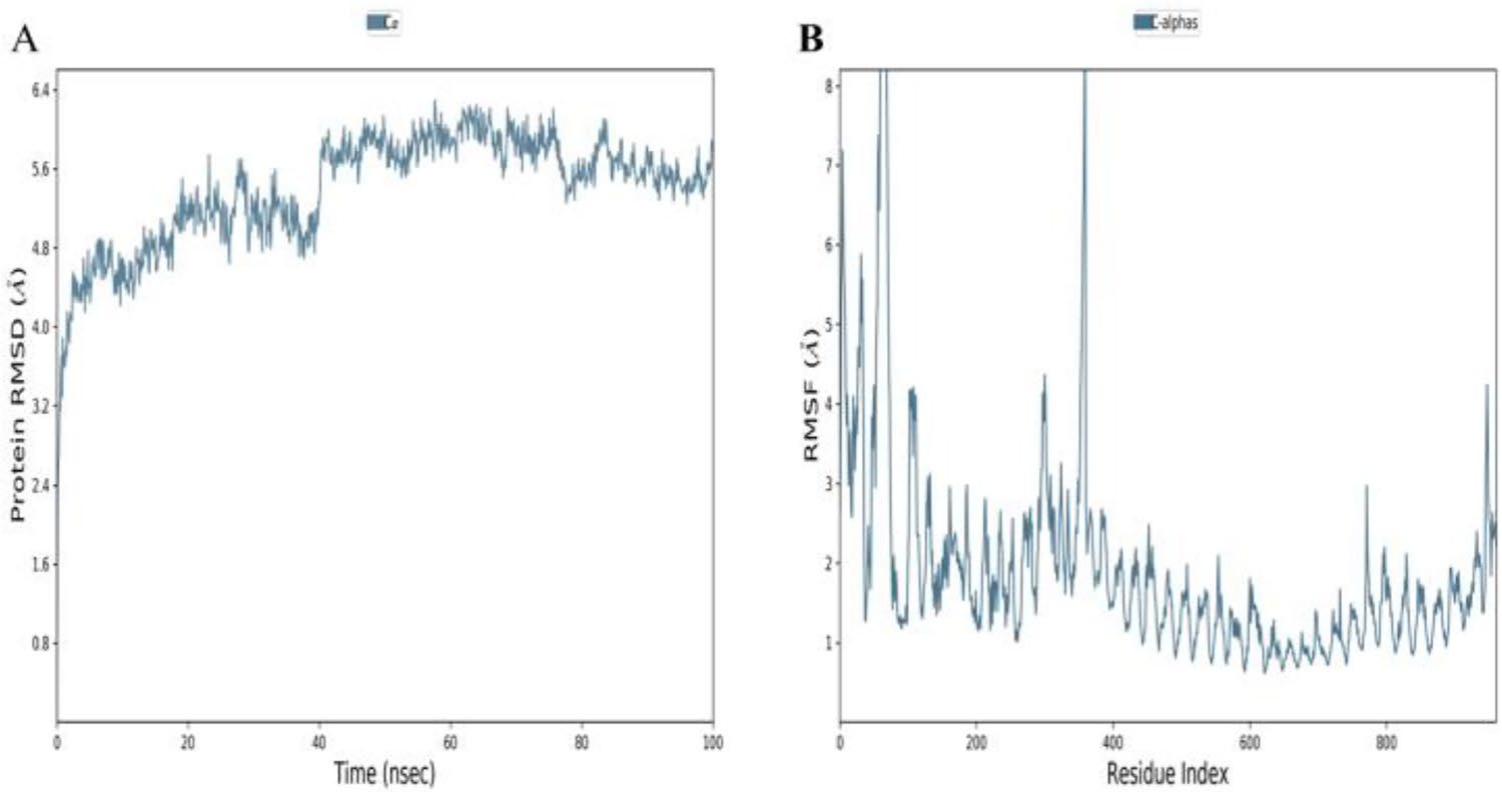
Molecular dynamics simulation-based statistical analysis to evaluate the inter-molecular stability and dynamics of the complexes: (**A**) Root Mean Square Deviation (RMSD) of the vaccine-receptor complex. (**B**) Root Mean Square Fluctuation (RMSF) of the vaccine-receptor complex.

Molecular Dynamics (MD) simulations are commonly used to analyze the dynamics and behavior of protein molecules at an atomic level. In MD simulations, the RMSD can be used to monitor the stability and conformational changes of a protein over time. To calculate the RMSD of a protein in an MD simulation, the trajectory of the protein's atomic coordinates is first extracted from the simulation output. Then, a reference structure is chosen, which can be either an experimental structure or an initial structure used in the simulation. The trajectory frames are superimposed onto the reference structure, and the RMSD is calculated for each frame. The RMSD values can be plotted as a function of time to visualize the protein's conformational changes and to determine the stability of the protein structure over the course of the simulation. Additionally, the RMSD values can be used to compare different simulations or to evaluate the accuracy of a computational model of a protein structure. In Figure 7 A, it has been shown that the vaccine-receptor docked complex was stable during the 100 ns time period of simulation. During 40 ns it was between 3.2 and 4.8 Å and after 40 ns until 100 ns, it remained between 4.8 Å and 5.6 Å which was quite acceptable. RMSD was slightly higher than an ideal value, but this was due to the loop region in the complex and it will not affect the function of the vaccine construct. RMSF (Root Mean Square Fluctuation) is a measure of the flexibility of a protein molecule. During an MD (Molecular Dynamics) simulation, the RMSF of a protein can be calculated by measuring the deviation of each atom in the protein from its average position over the course of the simulation. The RMSF profile provides a useful insight into the flexibility of different regions of the protein, which can help to understand the protein's function and how it interacts with other molecules. Similarly, Figure 7B revealed the structure compatibility during the 100 ns, but at some residues, RMSF was higher due to other secondary structures instead of alpha helices and beta sheets. RMSF was also in the range of an ideal value. It was between 3Å and 4Å.

### MMGBSA analysis

Using the MMGBSA, the binding free energy of the docked complex was determined and is shown in Table 7. ΔG_total_ value is typically used to calculate the protein-ligand complex's stability^63^. The stability of the complex is indicated by lower values of ΔG_total_, and vice versa. Van der Waals energy (EvdW), electrostatic energy (Eele), and GGB (electrostatic contribution to solvation-free energy via Generalized Born) are some of the protein-ligand interactions that contribute to the total binding free energy calculated using the MM/GBSA model.

**Table 7:** The Binding free energies of the complex calculated by applying MM/GBSA module.

Energy components	Energy value kcal/mol
ΔE_vdW_	−80.81
ΔE_ele_	−72.78
ΔE_GB_	37.55
ΔE_surf_	−7.73
ΔG_gas_	−152.67
ΔG_solv_	30.92
ΔG_total_	−122.85

### Immune simulation

**Figure 8:**
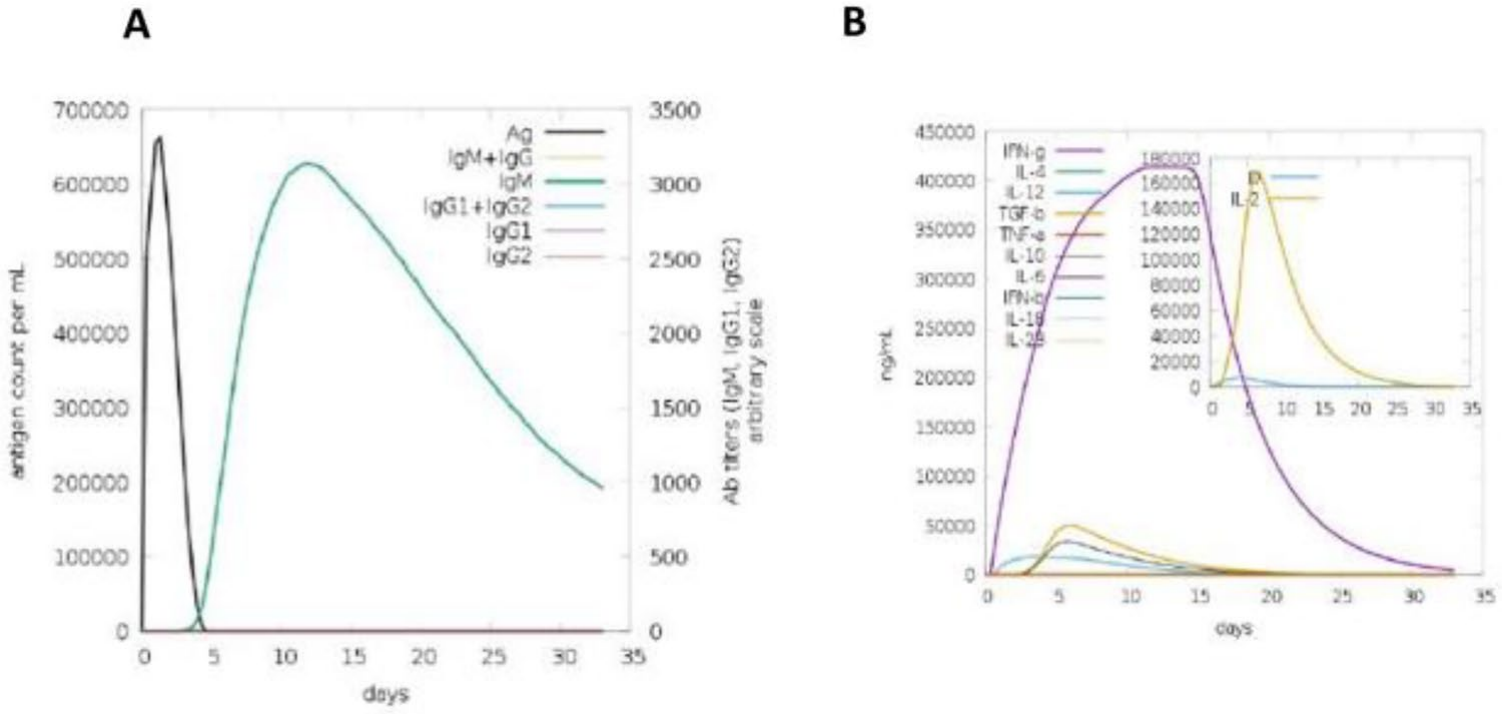
In silico immune simulation results obtained after administration of three injections of the vaccine. (**A**) Concentrations of immunoglobulins in proportion to antigen concentrations. (**B**) The production of various cytokines in response to the administration of c-ImmSim vaccine.

The immunogenic characteristics of potential *S. gallolyticus* vaccine candidate was ascertained using the C-IMMSIM server. Elevated IgM concentrations were found during the initial reaction, as predicted by the modeling results. Secondary and tertiary responses both demonstrated a typical increase in immunoglobulin activity, with accompanying antigen depletion (IgM, IgG + IgM, IgG1 + IgG2, and antibodies) (Figure 8A). Additionally, there were increased amounts of cytokines, including IFN- and IL-2, which are crucial for reducing cellular immunity and viral replication. The vaccine's immune-stimulating qualities ensured that it would be efficacious in human subjects (Figure 8B).

### Codon optimization and in silico cloning

**Figure 9:**
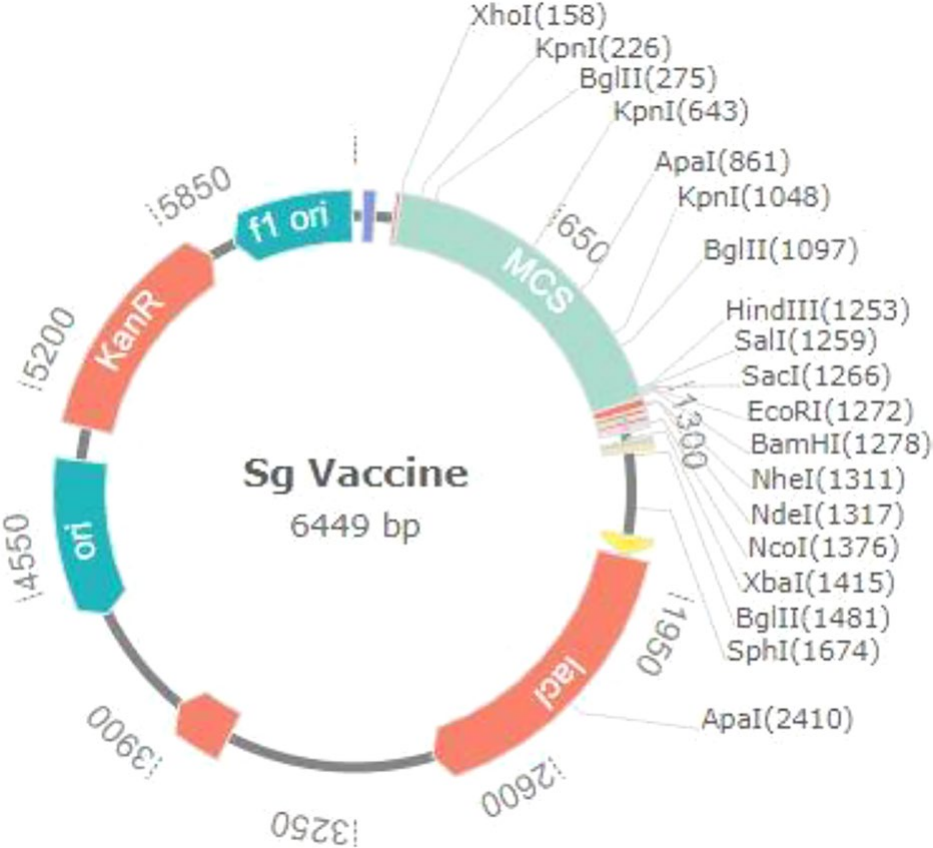
*S. gallolyticus* vaccine in silico restriction cloning into the pET28a (+) expression vector between between the XhoI and HindIII restriction sites. Vaccine construct is depicted as MCS in blue colour.

Because different codon usage patterns inhibit the translation of foreign genes, codon adaptation is the most efficient strategy for enhancing translational efficacy. The JCat program was used to optimize the codon usage of our developed vaccine in relation to the *E. coli* K12 strain. A CAI of 1 and a GC content of 48.3% in the optimized sequence indicated that it was effectively expressed in the *E. coli* host. As shown in Figure 9, the updated codon sequence from the vaccine construct was then inserted between the XhoI and HindIII restriction sites in the PET28a (+) E. coli expression vector. The size of the clone was 6449bp.

## Discussion

*S. gallolyticus* is one of the most common causes of bacterial endocarditis, accounting for up to 15-20% of all cases. *S. gallolyticus* endocarditis usually occurs in people with a history of congenital heart defects, heart valve disease, or intravenous drug use. Endocarditis caused by *S. gallolyticus* can be a serious and life-threatening condition with a mortality rate of 10-50%. Bacteria can enter the bloodstream through the mouth, gastrointestinal or dental surgery, or other sources of infection. *S. gallolyticus* is known to develop antibiotic resistance, which can complicate treatment and lead to treatment failure^64,65^. So, an effective vaccine against *S. gallolyticus* needs to be developed. Vaccines can help reduce the morbidity and mortality associated with this disease. Overall, the development of a vaccine against *S. gallolyticus*-associated endocarditis could have significant public health benefits, especially for people who already have heart disease and other risk factors. However, vaccine development requires extensive research, clinical trials, and regulatory approval, which can be a long and difficult process. The use of bioinformatics, reverse vaccinology, and immunoinformatics approaches has recently become an alternative method for developing vaccines against various pathogens^66,67^. Similar techniques have previously been used to propose vaccines for *Mycoplasma pneumoniae*^68^, *Klebsiella aerogenes*^69^, *Schistosomiasis*^70^, and *Campylobacter jejuni*^71^.

We developed multiple epitope-based vaccines against *S. gallolyticus* using pan-genome, immunoinformatics, subtractive proteomics, molecular docking, and simulation techniques. Seven well-known pathogenic *S. gallolyticus* strains were considered for vaccine development, and their core proteome analysis was performed. Because core proteins are present in all or most strains of the pathogen being targeted and their presence in vaccine development offers immune protection against a broader range of pathogen species, they are now being studied in the creation of vaccines^69^. The core proteome underwent a subtractive proteomics procedure to reveal essential, non-homologous, antigenic, immunogenic, and allergenic proteins for a vaccine. The presence of transmembrane helices is also a significant parameter for eliminating proteins. Since purifying proteins with multiple transmembrane helices is an arduous task, it appears wise to omit such proteins from the selection process. During the vaccine development process, this method checks that a prospective vaccine is suitable for experimental validation and that the proteins have little resemblance to human proteins to lessen the possibility of eliciting an autoimmune response. Two antigenic proteins: penicillin-binding protein and ATP synthase subunit were shortlisted as vaccine candidates. The target proteins anticipated CTL, HTL, and LBL epitopes. The majority of the work for the study was processed on IEDB tools, and the application was suitable and appropriate for thorough analysis, as indicated by Dhanda et al. (2019)^47^. Epitopes for the multi-epitope candidate were chosen for their antigenicity, immunogenicity, allergenicity, and toxicity. For a vaccine molecule to be effective, it must offer broad-spectrum defense against a variety of populations worldwide. Therefore, when developing an epitope-based subunit vaccination, it is necessary to identify the population fractions in the desired endemic regions according to HLA genotypic frequencies. The results showed that the chosen HTL and CTL epitopes covered a large portion of the endemic *S. gallolyticus* population.

Three linkers, successfully utilized by Israr et al.^72^, Ahtisham et al.^73^, Naveed et al.^74,75^, and Alizadeh et al.^76^, were used to adjoin the selected epitopes together. The AAY (Ala-Ala-Tyr) linker is the cleavage site for proteasomes in mammalian cells. Hence, the epitopes linked by the AAY linker are efficiently segregated within cells, lowering junctional immunogenicity. The AAY linker also improves the immunogenicity of the multi-epitope vaccine^77^. The lysosomal protease Cathepsin B targets the KK linker (lysine linker) in order to process the antigenic peptides and prepare them for presentation on the cell surface in an MHC-II restricted antigen presentation. Additionally, it is essential for lowering junctional immunogenicity because it prevents the formation of antibodies against the peptide sequence that occurs when individual epitopes are joined linearly^78^. Moreover, KK linkers boost immunogenicity^79^.GPGPG linkers have been shown to generate TH lymphocyte (HTL) responses, which is critical for a multi-epitope vaccination. Additionally, the GPGPG linker is an effective technique for reducing junctional immunogenicity, which restores the immunogenicity of specific epitopes. Livingston et al. empirically proved this using mouse models^80^. As an adjuvant, cholera toxin B subunit (CTB) was combined with the EAAAK linker. Due to its salt bridge coupled to lysine and glutamic acid, the EAAAK linker creates a stable helix structure and prevents protein domains from convergence^81^.CTB has been investigated as a conventional mucosal adjuvant that can increase the immunogenicity of vaccines^68,82^. Employing the adjuvant used by Mariam et al.^83^ and Tamalika et al.^84^ helped us achieve the desired stability while not affecting the antigenicity.

The structure of the developed vaccine solution was predicted to allow easy access to the host. Furthermore, the projected findings demonstrate that the *S. gallolyticus* vaccine is non-toxic, antigenic, and non-allergenic. The outcomes for each of the three categories aligned with the findings of Naveed et al. (2021), who conducted antigenicity analyses all along the way^75^. Similar methods were used in this study, and the final epitopes were selected by processing each epitope's unique antigenicity and allergenicity. A good vaccine must have antigenic qualities that are required to elicit the host's immunological response, as well as the ability to develop long-lasting adaptive immunity through the antigen. Solubility is a crucial development characteristic because the vaccine will be delivered in a water environment inside the host body. It has been found that low solubility subunit vaccines are ineffective at producing significant amounts of viral proteins. Hence, creating vaccines with high solubility is crucial for various functional and biochemical studies. The vaccine design was analyzed to be soluble upon expression, meaning that it would have easy access to the host.

TLRs are essential for innate immune system activation and viral particle identification. TLRs have the potential to be an important target for the creation of vaccines and disease prevention^85^. The precise binding affinities and interactions of the multi-epitope final subunit vaccine design against TLR4 were investigated using molecular docking and simulation studies. During docking, a significant number of H-bonds were observed, as well as minor fluctuations during MD simulations. These findings suggest that the vaccine can effectively bind to TLR4.

Codon optimization is required in prokaryotic hosts for the production of eukaryotic proteins because all synonymous codons within a codon family do not express diverse proteins at the same rate in *E.coli*^67^. Therefore, to increase protein expression levels, codon optimization was carried out in *E. coli* strain K12. Hence, the codon compatibility index (1.0) and the GC content (48%) were determined, indicating a significant likelihood of protein expression. Furthermore, immunological simulation of the designed vaccine produced promising findings in terms of cellular and humoral immune response. According to the bioinformatics analysis of the vaccine design, this candidate may be particularly effective against *S. gallolyticus*. Immunoinformatics/computational methodologies were developed on the basis of experimental procedures to create raw data for research objectives. The consistency and proficiency of computer algorithms limit the accuracy of immunoinformatics predictions. Therefore, in vitro and in vivo experiments are needed to confirm the real potential of the proposed anti-*S. gallolyticus* vaccine.

## Conclusion

The development of a proteomics-based approach to identifying vaccine targets against antibiotic-resistant *S.gallolyticus* is a promising avenue for combating this difficult pathogen. A comprehensive analysis of the bacterial proteomes of seven strains revealed two antigenic proteins as vaccine candidates and identified potential epitopes, laying the groundwork for the development of a multi-epitope vaccine. A safe and latent vaccine that may trigger humoral, cellular, and innate immune responses was developed using immunoinformatics and molecular docking approaches. By targeting multiple epitopes, such a vaccine could improve immune recognition and address the issue of antigenic variation, potentially providing broad protection against various strains of *S. gallolyticus*. This novel strategy has great promise for combating the growing threat of antibiotic resistance and improving public health outcomes. However, more research and clinical trials are required to validate the efficacy and safety of this vaccine candidate before it can be widely used as a preventive measure against *S. gallolyticus* infections.

### Supplementary Information

Supplementary Information.

## Data Availability

The datasets analyzed during the current study are available in the NCBI database and IDs of the datasets are mentioned in Additional File 1: Table S1.
