# Supplementary material for: Proteomics-based vaccine targets annotation and design of multi-epitope vaccine against antibiotic-resistant Streptococcus gallolyticus
Source: Sci Rep. 2024 Feb 28;14:4836. doi: 10.1038/s41598-024-55372-3 (PMC10901886; doi:10.1038/s41598-024-55372-3)
Supplement: Supplementary file 1 — Supplementary Information. [file 41598_2024_55372_MOESM1_ESM.docx]

***Table S1*** Details of [*Streptococcus gallolyticus*](https://www.ncbi.nlm.nih.gov/genome/2742?genome_assembly_id=906560) Strains

| Organism Name | Organism Groups | Strain | BioSample | BioProject | Assembly | Size | GC% | CDS | Release Date |
| --- | --- | --- | --- | --- | --- | --- | --- | --- | --- |
| [Streptococcus gallolyticus](https://www.ncbi.nlm.nih.gov/genome/2742?genome_assembly_id=906560) | Bacteria;Terrabacteria group;Bacillota | [FDAARGOS_755](https://www.ncbi.nlm.nih.gov/genome/2742?genome_assembly_id=906560) | [SAMN11056470](https://www.ncbi.nlm.nih.gov/biosample/SAMN11056470) | [PRJNA231221](https://www.ncbi.nlm.nih.gov/bioproject/PRJNA231221) | [GCA_013267695.1](https://www.ncbi.nlm.nih.gov/assembly/GCA_013267695.1) | 2.26 | 37.70 | [2,155](https://www.ncbi.nlm.nih.gov/genome/browse/#!/proteins/2742/906560%7CStreptococcus%20gallolyticus/) | 04-Jun-2020 |
| [Streptococcus gallolyticus](https://www.ncbi.nlm.nih.gov/genome/2742?genome_assembly_id=906560) | Bacteria;Terrabacteria group;Bacillota | [XH2168](https://www.ncbi.nlm.nih.gov/genome/2742?genome_assembly_id=2121514) | [SAMN31395130](https://www.ncbi.nlm.nih.gov/biosample/SAMN31395130) | [PRJNA892746](https://www.ncbi.nlm.nih.gov/bioproject/PRJNA892746) | [GCA_027474865.2](https://www.ncbi.nlm.nih.gov/assembly/GCA_027474865.2) | 2.39 | 37.70 | [2,266](https://www.ncbi.nlm.nih.gov/genome/browse/#!/proteins/2742/2121514%7CStreptococcus%20gallolyticus/) | 28-Dec-2022 |
| [Streptococcus gallolyticus](https://www.ncbi.nlm.nih.gov/genome/2742?genome_assembly_id=906560) | Bacteria;Terrabacteria group;Bacillota | [ICDDRB-NRC-S1](https://www.ncbi.nlm.nih.gov/genome/2742?genome_assembly_id=260392) | [SAMN04348601](https://www.ncbi.nlm.nih.gov/biosample/SAMN04348601) | [PRJNA306242](https://www.ncbi.nlm.nih.gov/bioproject/PRJNA306242) | [GCA_001477575.1](https://www.ncbi.nlm.nih.gov/assembly/GCA_001477575.1) | 2.05 | 37.70 | [1,919](https://www.ncbi.nlm.nih.gov/genome/browse/#!/proteins/2742/260392%7CStreptococcus%20gallolyticus/) | 22-Dec-2015 |
| [Streptococcus gallolyticus](https://www.ncbi.nlm.nih.gov/genome/2742?genome_assembly_id=906560)  [UCN34](https://www.ncbi.nlm.nih.gov/genome/2742?genome_assembly_id=172621) | Bacteria;Terrabacteria group;Bacillota | [UCN34](https://www.ncbi.nlm.nih.gov/genome/2742?genome_assembly_id=172621) | [SAMEA2272396](https://www.ncbi.nlm.nih.gov/biosample/SAMEA2272396) | [PRJEA34729](https://www.ncbi.nlm.nih.gov/bioproject/PRJEA34729) | [GCA_000027185.1](https://www.ncbi.nlm.nih.gov/assembly/GCA_000027185.1) | 2.35 | 37.60 | [2,229](https://www.ncbi.nlm.nih.gov/genome/browse/#!/proteins/2742/172621%7CStreptococcus%20gallolyticus%20UCN34/) | 10-Feb-2010 |
| [Streptococcus gallolyticus](https://www.ncbi.nlm.nih.gov/genome/2742?genome_assembly_id=906560) [ATCC 43143](https://www.ncbi.nlm.nih.gov/genome/2742?genome_assembly_id=172623) | Bacteria;Terrabacteria group;Bacillota | [ATCC 43143](https://www.ncbi.nlm.nih.gov/genome/2742?genome_assembly_id=172623) | [SAMD00060983](https://www.ncbi.nlm.nih.gov/biosample/SAMD00060983) | [PRJDA62517](https://www.ncbi.nlm.nih.gov/bioproject/PRJDA62517) | [GCA_000270145.1](https://www.ncbi.nlm.nih.gov/assembly/GCA_000270145.1) | 2.36 | 37.50 | [2,244](https://www.ncbi.nlm.nih.gov/genome/browse/#!/proteins/2742/172623%7CStreptococcus%20gallolyticus%20subsp.%20gallolyticus%20ATCC%2043143/) | 28-May-2011 |
| [Streptococcus gallolyticus](https://www.ncbi.nlm.nih.gov/genome/2742?genome_assembly_id=906560)  [ATCC BAA-2069](https://www.ncbi.nlm.nih.gov/genome/2742?genome_assembly_id=172622) | Bacteria;Terrabacteria group;Bacillota | [ATCC BAA-2069](https://www.ncbi.nlm.nih.gov/genome/2742?genome_assembly_id=172622) | [SAMEA2272408](https://www.ncbi.nlm.nih.gov/biosample/SAMEA2272408) | [PRJEA63179](https://www.ncbi.nlm.nih.gov/bioproject/PRJEA63179) | [GCA_000203195.1](https://www.ncbi.nlm.nih.gov/assembly/GCA_000203195.1) | 2.38 | 37.60 | [2,251](https://www.ncbi.nlm.nih.gov/genome/browse/#!/proteins/2742/172622%7CStreptococcus%20gallolyticus%20subsp.%20gallolyticus%20ATCC%20BAA-2069/) | 03-Mar-2011 |
| [Streptococcus gallolyticus](https://www.ncbi.nlm.nih.gov/genome/2742?genome_assembly_id=906560)  [TX20005](https://www.ncbi.nlm.nih.gov/genome/2742?genome_assembly_id=1648582) | Bacteria;Terrabacteria group;Bacillota | [TX20005](https://www.ncbi.nlm.nih.gov/genome/2742?genome_assembly_id=1648582) | [SAMN17138288](https://www.ncbi.nlm.nih.gov/biosample/SAMN17138288) | [PRJNA684625](https://www.ncbi.nlm.nih.gov/bioproject/PRJNA684625) | [GCA_019021805.1](https://www.ncbi.nlm.nih.gov/assembly/GCA_019021805.1) | 2.26 | 37.70 | [2,156](https://www.ncbi.nlm.nih.gov/genome/browse/#!/proteins/2742/1648582%7CStreptococcus%20gallolyticus%20subsp.%20gallolyticus%20TX20005/) | 24-Jun-2021 |

***Table S2*** Toxicity Prediction of Final Epitopes Predicted by ToxinPred

| Epitopes | Toxicity |
| --- | --- |
| **CTL Epitopes** | |
| ILAVFLFFV | Non-Toxin |
| FVFVINFAI | Non-Toxin |
| FQNEAQGTY | Non-Toxin |
| MLEPQFISQ | Non-Toxin |
| ASAEDGGGY | Non-Toxin |
| GSFLLLLVL | Non-Toxin |
| LLLVLIKIF | Non-Toxin |
| **HTL Epitopes** | |
| ERVGQNLMILAVFLF | Non-Toxin |
| VFLFFVFVINFAIII | Non-Toxin |
| ISNDIDGAEAAREKA | Non-Toxin |
| **LBL Epitopes** | |
| NRERVGQNLMILAVFL | Non-Toxin |
| AIIIGTDTKFGHNLST | Non-Toxin |
| ISQIYDPNTNSARVAT | Non-Toxin |
| TGVFKAREEKISNDID | Non-Toxin |
| DGAEAAREKAEALAAK | Non-Toxin |
